# Shikonin induces cell autophagy via modulating the microRNA -545-3p/guanine nucleotide binding protein beta polypeptide 1 axis, thereby disrupting cellular carcinogenesis in colon cancer

**DOI:** 10.1080/21655979.2021.2024638

**Published:** 2022-02-22

**Authors:** ZhiWei Hu, XinDong Zhou, DeQiang Zeng, JiaJun Lai

**Affiliations:** Department of Gastrointestinal Surgery, The Yuebei People’s Hospital of ShaoGuan, ShaoGuan, GuangDong, China

**Keywords:** Shikonin, colorectal cancer, autophagy, microrna-545-3p, guanine nucleotide binding protein beta polypeptide 1

## Abstract

Shikonin (SHK), a major component of shiverweed, was provided with anti-tumor effects via multiple targets and signal pathways. Nevertheless, the specific mechanism of its function in colorectal cancer (CRC) still needed to be further explored. The study was designed to examine the role of SHK in CRC and its specific mechanism on the cell tumor behavior of CRC. Collection of clinical samples was performed, and test of microRNA (miR)-545-3p and guanine nucleotide-binding protein beta polypeptide 1 (GNB1) in the samples was conducted; Selection of CRC cell line was exerted, and examination of miR-545-3p and GNB1 was performed; After treatment of shikonin (SHK), correlated plasmids were transfected, test of cell advancement was performed. Test of the protein of autophagy-correlated proteins light chain 3-II/light chain 3I and p63 was performed. The interaction of miR-545-3p with GNB1 was explored, and the action of SHK in *vivo* was tested. SHK repressed the advancement of SW480 cells with elevated apoptosis and autophagy and the cells quantities in G0/G1 phase. MiR-545-3p was elevated in CRC. SHK boosted miR-545-3p, repression of miR-545-3p or augmentation of GNB1 was able to turn around the function of SHK on CRC, and GNB1 was the target gene of miR-545-3p.All in all, SHK stimulates apoptosis and autophagy in CRC via miR-545-3p/GNB1 signaling axis, firstly demonstrating the regulatory mechanism of SHK in CRC via miR-545-3p/GNB1 axis.

## Graphical Abstract

**Figure:**
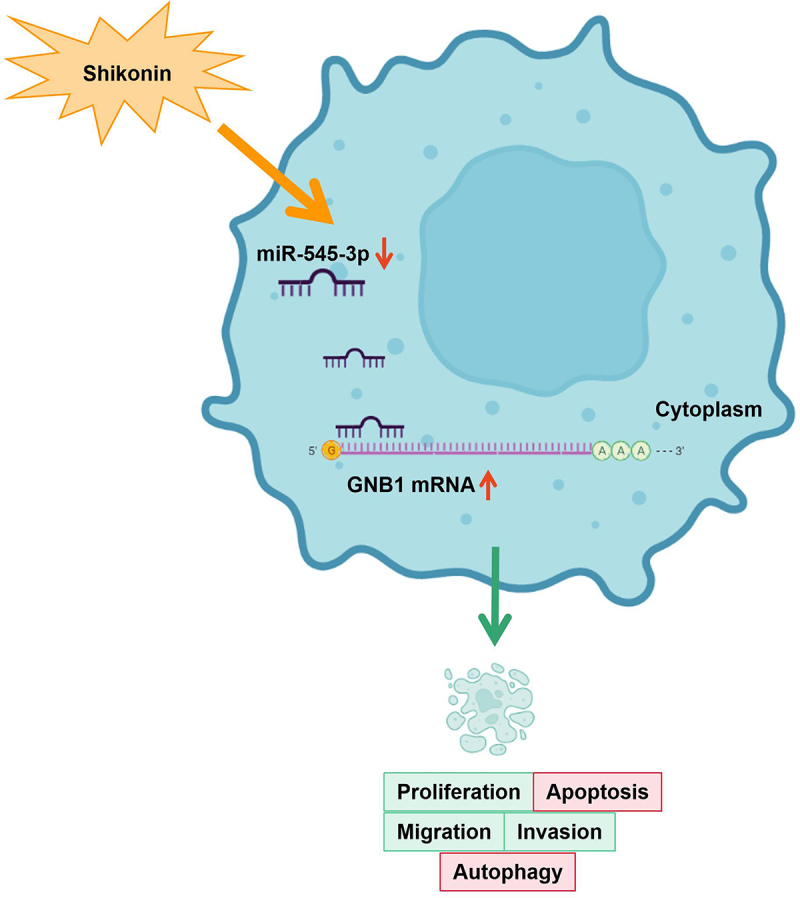

## 1 Introduction

Colorectal cancer (CRC) is a prevalent cancer with an elevated fatality rate [1]. With constant new breakthroughs in surgery and molecular-targeted treatment in the past few years, the therapeutic function of CRC has increasingly elevated, but the 5-year survival rate of CRC patients is continuously declined [2]. Presently, the best method for early diagnosis of CRC is colonoscopy. Nevertheless, it is hard to be broadly implied due to the limitation of its invasive examination method, diet, and cost, while the accuracy and sensitivity of other examination methods still are provided with shortcomings [3]. Consequently, it is urgent to develop diagnosis and treatment methods with novel noninvasive, low side-effect and high accuracy to enhance the survival rate and prognosis of patients [4].

Discovery of anticancer drugs from natural products has been going on for decades. Many important anticancer drugs have been successfully isolated from plants, such as vincristine and vincristine isolated from Malagasy catharanthus roseus, and catharanthus roseus. With the development of knowledge in the field of chemistry, the discovery of anticancer drugs is not limited to the isolation and direct application of bioactive compounds, but also extends to the structural modification of these compounds to improve the pharmacokinetic and pharmacodynamic profiles [5–7]. Studies have been clarified that shikonin (SHK) is a natural naphthoquinone compound with anti-tumor and anti-cancer effects [8]. For instance, SHK suppresses oral squamous cell carcinoma cell advancement via modulating the microRNA(miR)-9/RECK axis [9]. SHK suppresses hepatocellular carcinoma (HCC) cells via silence of microRNA (miR)-92a [10]. Study clarifies that SHK is able to repress CRC cell proliferation [11]; nevertheless, the molecular mechanism in which SHK impacts CRC via modulating microRNA (miRNA) was not explored.

MiRNA, a type of small non-coding RNA, is provided with the functions of silencing RNA and modulating gene [12]. Numerous studies have clarified that aberrant manifestation of miRNA is linked with CRC progression [13]. For instance, miR-574-3p stimulates CRC cell apoptosis via directly targeting CCND2 [14]. Studies have elucidated that miR-545-3p is linked to certain cancers. For instance, miR-545-3p is silenced in HCC, and HCC cell advancement is boosted via silence of metallothionein 1 M (MT1M) [15]. MiR-545-3p also manifests low expression in oral squamous cell carcinoma [16], lung cancer [17], and cervical cancer [18]. Nevertheless, limited reports are presented about the role of miR-545-3p in CRC.

G protein subunit β1/guanine nucleotide-binding protein β1 (GNB1) encodes the Gβ subunit of a heterotrimeric G protein complex which also covers Gα and Gγ subunits and is capable of transducing intracellular signaling cascades [19]. Few studies were on GNB1 so far, and it was primarily correlated with human neurological diseases. Meanwhile, few reports on the action of GNB1 in cancer as well. Foregoing studies have clarified that GNB1 is silenced in clear cell renal cell carcinoma (CCRCC), and linked with the deterioration of CCRCC and vascular endothelial growth factor (VEGF) signal transduction in CCRCC [20]. Nevertheless, the mechanism of GNB1 in CRC remains to be uncovered.

In this study, it was aimed to investigate the role and downstream molecular mechanisms of SHK in CRC. *In vitro* and *vivo* experiments were carried out by collecting CRC clinical samples, CRC cell lines and nude mice to analyze the effects of SHK on autophagy, apoptosis and tumor growth of CRC cells. It was hypothesized that SHK induced apoptosis and autophagy of CRC cells through miR-545-3p/GNB1 signaling axis. This study will provide a new direction for CRC treatment.

## 2 Method

### 2.1 Tissue specimens

A total of 67 patients with CRC underwent surgical resection in The Yuebei people’s Hospital of ShaoGuan. No reception of radiotherapy or chemotherapy was in the patients prior to surgery. All subjects offered written informed consent prior to collection and adoption of their specimens. Two clinical pathologists diagnosed the histopathological type of CRC. The protocol has been authorized via the Human Subjects Protection Committee of The Yuebei people’s Hospital of ShaoGuan.

### 2.2 Cell culture

Human colonic epithelial cell-line NCM460 and CRC cell line HCT116, SW480, LoVo, HT29 cells were offered (all Chinese Academy of Sciences, Shanghai, China). The medium for all cells was 90% Roswell Park Memorial Institute 1640 [21]. Culture of the cells was performed. When the cells grew logarithmically, the experiment was started. SHK was conducted (all Selleck Chemicals, Houston, TX, USA), and storing was performed. Dilution of the storage solution was to the required concentration prior to each experiment. Treatment of the cells was with diverse concentrations of SHK.

### 2.3 Cell transfection

Small interring RNA targeting GNB1 (si-GNB1) for miR-545-3p mimics or inhibitors, oe-GNB1 and their respective negative controls were chemically synthesized (Guangzhou RiboBio Co., Ltd., Guangzhou, China). Placing of SW480 cells was in a 6-well plate at a density of 3 × 10^5^ cells per well, and cell density was determined by cell densitometer (Shanghai Farun Scientific Instrument Co., Ltd., Shanghai, China), and transfection was with Lipofectamine 2000 (Invitrogen) on the grounds of the manufacturer’s instructions. Collection of the cells was for follow-up experiments.

## 2.4 3-(4, 5-dimethylthiazol-2-yl)-2, 5-diphenyltetrazolium bromide (MTT)

Test of cell viability was performed. Seeding of the cells was uniformly in a 96-well plate with 2 × 10^3^ cells per well with incubation. Then treatment of each column was with diverse concentrations of SHK or control medium, and addition of MTT solution was to each well. Measurement of the absorbance of individual wells was with a microplate reader (Bio-Rad, Sunnyvale, CA, USA) at 570 nm behind incubation [22].

### 2.5 Cell cycle analysis

Culture of SW480 cells was in 6-well plates and treatment was with SHK at diverse concentrations. The cell cycle experiments were performed adopting the cell cycle staining kit on the grounds of the manufacturer’s instructions. Briefly, after incubation with 0.05 mg 0.1% sodium citrate (involving propidium iodide (PI)) and 50 g RNase, analysis of the cell was with FACSCalibur flow cytometry (FC500, Beckman Coulter, FL, USA). Analysis of the cell proportion (percentage) in G0/G1, S and G2/M phases of the cell cycle was with multi-cycle AV DNA analysis software (Phoenix Flow Systems, Inc.) [23].

### 2.6 Transwell migration and invasion

After incubation, trypsinization of the transfected cells was with 0.25% trypsin, and resuspension was in serum-free medium. Adjustment of the concentration of the cell suspension was to 5 × 10^5^ cells/mL. A Transwell chamber was adopted with the cell migration test (pore size was 8 μM; BD Biosciences), while the cell invasion test was performed adopting a matrix-coated chamber (BD Biosciences). Loading of the apical compartment was with 200 μL cell suspension, and loading of the basolateral compartment was with 500 μL complete medium covering 10% fetal bovine serum. Behind 24 h, collection of cells having migrated or invaded into the pores was conducted. Fixation of cell advancement was with 5% glutaraldehyde, and staining was with 0.1% crystal violet. Photograph of the cells was with an optical microscope (Olympus, Tokyo, Japan) [24].

### 2.7 Analysis of cell apoptosis

Determination of the relative number of apoptotic cells was with the Annexin V Fluorescein Isothiocyanate (FITC) Apoptosis Detection Kit (Biolegend, San Diego, CA, USA). Shortly, obtainment of the transfected cells was conducted after 48 h of culture, and centrifugation was performed. Resuspension of the collected cells was in the combining buffer, and staining was with 5 μL annexin V-FITC and 10 μL PI with quantification of the apoptotic cells [25].

### 2.8 Reverse transcription quantitative polymerase chain reaction (RT-qPCR)

Total RNA was isolated from adherent cells using TRIzol reagent. Thermo Nano Drop 2000 was applied to detect the concentration and purity of total RNA, and sulfate polyacrylamide gel electrophoresis Agilent-2100 was used to detect the integrity of total RNA. According to manufacturer’s instructions, application of QuantiTect® reverse transcription kit was to reverse transcribe 500 ng total RNA to complementary DNA (cDNA). In short, 1 μg template RNA, and 2 μL 7 × DNA Wipeout buffer were mixed with diethyl carbonate-treated water to a final volume of 14 μL and incubated at 42°C for 2 min, then they were cooled on ice and then added with 1 μL Quantiscript reverse transcriptase, 4 μL 5 × Quantiscript RT buffer and 1 μL 1 × RT primer mixture, and incubated at 42°C for 15 min. Finally, the tubes were incubated at 95°C for 3 min to inactivate Quantiscript reverse transcriptase. The cDNA samples were stored at −20°C until use.

Real-time quantitative PCR was performed in the LightCycler 480 real-time PCR system (Roche Applied Science, Mannheim, Baden-Wuerttemberg, Germany). The primers used for PCR were shown in Table 1. Final volume of reaction mixture was 10 μL, each primer was 1 μmol/L, 2 × SYBR Green Master was 5 μL, cDNA was 4 μL. The conditions of the PCR scheme were as follows: The initial template was activated at 95°C for 5 min, followed by 50 cycles at different temperatures/times (95°C 15 s, 65°C 30 s and 72°C). Gene expression levels normalized Ct values to internal control glyceraldehyde-3-phosphate dehydrogenase (GAPDH) and were calculated as a percentage relative to untreated cells [26]. Table 1 manifests the primers.

**Table 1.** Primer sequence

Genes	Primer sequences (5′ – 3′)
MiR-545-3p	F: 5ʹ -TGCGCTCAGCAAACATTTATTG-3ʹ
	R: 5ʹ -CCAGTGCAGGGTCCGAGGTATT-3ʹ
U6	F: 5ʹ -CTCGCTTCGGCAGCACATATACTA-3ʹ
	R: 5ʹ -ACGAATTTGCGTGTCATCCTTGCG-3ʹ
GNB1	F: 5′-AGGGGTAAGGGAGCAGAG-3′
	R: 5′ -GCAGCAGTAGTGGCTTCTCC-3′
GAPDH	F: 5ʹ -CCATGGAGAAGGCTGGGG-3’
	R: 5ʹ -CAAAGTTGTCATGGATGACC-3’

**Note: F: Forward; R: Reverse**

### 2.9 Western blot

Extraction of the total protein sample from the cell line was with radio-immunoprecipitation assay lysis buffer; Determination of the protein concentration was with the bicinchoninic acid protein assay kit (all from Beyotime Institute of Biotechnology). An equal amount of protein sample (30 g) was separated on 10% sulfate polyacrylamide gel electropheresis and transfer was to polyvinylidene fluoride membrane (EMD Millipore). Seal of the cell membrane was with 5% (w/v) skim milk in Tris buffered saline covering 0.2% Tween-20. Incubation of primary antibodies of anti-GNB1 (1: 1000; 10,247-2-AP; Proteintech), LC3 (1: 1000; 2775), p62 (1: 1000; 5114), and GAPDH (1: 1000; 2118; Cell Signaling Technology) was performed; Adoption of goat anti-rabbit IgG-horseradish Peroxidase secondary antibody (MBS435036; MyBioSource) was performed, and visualized analysis was via enhanced chemiluminescence detection system (Santa Cruz Biotechnology, Inc.) [27].

### 2.10 The luciferase activity assay

Prediction of the combination of miR-545-3p and GNB1 was via TargetScan7.1 (http://www.targetscan.org/vert_71/). Clone of the wild-type (WT) or mutant (MUT) 3ʹ-untranslated region (3ʹ-UTR) of GNB1 covering miR-545-3p binding site was into the pmirGLO luciferase reporter vector (LMAI Bio, LM-1439) adopting restriction enzymes. Co-transfection of SW480 cells was with wide-type/mutant 3ʹURT and miR-545-3p for 48 h adopting Lipofectamine 2000 reagent (Invitrogen, 11,668,019). Lysis of the cells was performed, and the luciferase activity was assessed [28].

### 2.11 RNA immunoprecipitation (RIP)

RIP was conducted adopting Magna RIP RNA binding protein immunoprecipitation kit (Sigma, CA, USA). Transfection of SW480 was via adopting miR-545-3p-biotin or non-sense control (NC)-biotin. Lysis of the cells was with RIP lysis buffer. Adoption of A/G magnetic beads and anti-biotin ligation was to repress miR-545-3p-biotin immunoprecipitation. Test of GNB1 and miR-545-3p in the sediment was conducted after recovering the antibody from the protein beads [29].

### 2.12 Construction of Xenotransplantation

Performance of animal experiments was on the grounds of the procedures authorized via the Animal Care and Use Committee of Performance of animal experiments was on the grounds of the procedures authorized via the Animal Care and Use Committee of The Yuebei people’s Hospital of ShaoGuan and the guidelines for the Care and Use of Experimental Animals of National Institutes of Health. Specific sterile-grade BALB/c nude mice (6 weeks) were conducted (all Beijing Vital River Laboratory Animal Technology Co., Ltd., Beijing, China). Division of the selected mice was randomly into 2 groups (the control and the SHK). Implantation of SW480 cells (5 × 10^6^) in 100 μL of phosphate buffered saline was subcutaneously into the right side of nude mice (Vital River Laboratory, Beijing, China). Saline SHK5 g/kg was injected intraperitoneally once a day with or without it. Tumor development was monitored in line with volume = 1/2 (length × width ^2^). After injection 28 d, the mice were euthanatized via neck dislocation after 2% isoflurane deep anesthesia for subsequent experiments [30].

### 2.13 TdT-mediated dUTP-biotin nick end-labeling (TUNEL) analysis

Dewaxing was with xylene and ethanol gradient, and incubation was with proteinase K (20 μg/mL dissolved in Tris/HCI, pH 7.4 8.0). The methods refer to the instructions of TUNEL Apoptosis Detection Kit. The positive staining was brownish yellow and situated in the nucleus. Selection of 10 high-power fields for each section, count of 200 cells was in each field, and calculation of the proportion of positive cells and the average was performed. N = 3.

### 2.14 Statistical analysis

Analysis of the data was adopting SPSS 21.0 (SPSS, Inc, Chicago, IL, USA) statistical software. After the Kolmogorov–Smirnov test, the data were normally distributed, and the results were manifested as the mean ± standard deviation (SD). Two-group comparison was adopting t test, the comparison between multiple was adopting one-way analysis of variance (ANOVA), and the pairwise comparison behind ANOVA analysis was exerting the least distinct difference (Fisher’s least significant difference t test, LSD-t). Presentation of the enumeration data was via rate or percentage, and comparative analysis was with the chi-square test. *P* < 0.05 manifested distinct differences.

## 3 Results

In the study, via *in vitro* and *in vivo* experiments, analysis was predicted that SHK might play a key role in CRC through the miR-545-3p/GNB1 axis. Therefore, it aimed to investigate the biological role and downstream molecular mechanisms of SHK in CRC. The data manifested miR-545-3p was down-regulated, but GNB1 was up-regulated in CRC. SHK repressed the migration and invasion, but promoted apoptosis and autophagy of CRC cells, while miR-545-3p/GNB1 axis was downstream of SHK, which reversed the effect of SHK on CRC cells to some extent. In conclusion, the results reveal SHK induces apoptosis and autophagy in CRC cells through miR-545-3p/GNB1 signaling axis, which may provide a novel approach for the treatment of CRC.

### 3.1 MiR-545-3p is silenced in CRC and linked with CRC progress

**Figure 1.**
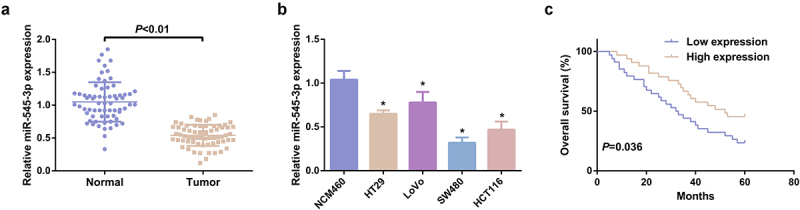
MiR-545-3p is silenced in CRC. (A) Test of the relativity of miR-545-3p in paired CRC and adjacent normal tissues was via reverse transcription quantitative PCR (n = 67). (B) Examination of miR-545-3p in CRC cell lines and NCM460 cells was via qPCR; (C) Kaplan Meier survival analysis manifested elevated miR-545-3p was linked with poor prognosis in CRC patients. * *P*< 0.05 versus the NCM460 cells. The data in the figures were all measurement data in the form of mean ± SD.

In this research, determination of miR-545-3p was in 67 pairs of CRC and normal tissue samples. MiR-545-3p was distinctively silenced in CRC tissues versus the normal samples (Figure 1a). The CRC cell line was selected, prior to which, the tumorigenicity of NCM460 cells was evaluated and preliminary identification manifested NCM460 cells did not grow in soft agar, indicating that it is nontumorigenic [31]. The qPCR experiment clarified miR-545-3p expression was down-regulated in CRC cells vs. NCM460 cells (Figure 1b). Additionally, the prognosis of miR-545-3p was assessed. CRC cases with declined miR-545-3p were provided with poor overall survival versus the CRC cases with elevated miR-545-3p (Figure 1c). Division of CRC patients was into the elevated (n = 33) and the declined (n = 34) in line with median of miR-545-3p to figure out the association of miR-545-3p with the clinicopathological features of patients. Distinct associations were of miR-545-3p with lymph node metastasis, tumor node metastasis (TNM) staging and distant metastasis (Table 2). Other clinicopathological parameters (such as tumor size, gender, age, smoking history, and alcohol consumption) were not distinctly correlated with miR-545-3p. These data confirmed the expression and correlation of miR-545-3p in CRC for the first time, which has certain novelty.

**Table 2.** Association of miR-545-3p and clinicopathological features of CRC patients

		MiR-545-3p		
Variables	Cases (n = 67)	The declined (n = 34)	The elevated (n = 33)	*P*
Sex				0.224
Male	35	15	20	
Female	32	19	13	
Age, years				0.807
Less than 60	30	16	14	
60 or less	37	18	19	
Smoking history				1.000
Yes	34	17	17	
No	33	17	16	
Alcohol consumption				0.806
Yes	40	21	19	
No	27	13	14	
Tumor size, cm				0.141
5 or more	38	16	22	
Less than 5	29	18	11	
Lymph node metastasis				0.002
Yes	43	28	15	
No	24	6	18	
Distant metastasis				0.012
Yes	17	13	4	
No	50	20	30	
TNM stage				<0.001
I/II	44	15	29	
III/IV	23	19	4

### 3.2 SHK represses the advancement with facilitated autophagy of CRC cells

**Figure 2.**
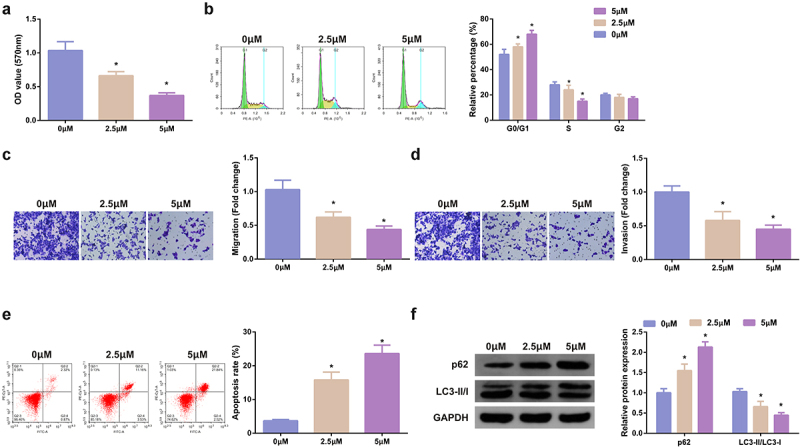
SHK suppresses the progression with facilitated autophagy of CRC cells. (A) Determination of the influence of SHK on cell viability was via MTT method. (B) Treatment of the cells was with different concentrations of SHK for 24 h, and test of the cell cycle was via flow cytometry. (C, D) Examination of cell migration and invasion was via Transwell. (E) Detection of cell apoptosis was via Flow cytometry. (F) Analysis of autophagy-correlated protein was via Western blot, GAPDH presented as load control. Treatment of SW480 cells was with diverse concentrations of SHK medium. N = 3; * *P* < 0.05 versus the 0 mg/mL concentration of SHK. The data in the figures were all measurement data in the form of mean ± SD.

Treatment of the cells was with diverse concentrations of SHK, and determination of the influence of diverse concentrations of SHK was on CRC cell viability. The cell viability of SW480 was declined with the augmentation of SHK concentration, as proved in Figure 2a. Test of the cell cycle of SW480 cells was performed and the proportion of cells in each group was observed, which manifested that the G0/G1 of SW480 cells was distinctively elevated versus the control after the addition of SHK, implying that SHK treatment was able to stimulate the SW480 cells to remain in the G0/G1 phase (Figure 2b). Additionally, SHK was available to suppress the migration and invasion of SW480 cells (Figure 2c, d). SHK dramatically boosted SW480 cell apoptosis (Figure 2e). Studies have clarified that autophagy was nearly associated with the occurrence and development of tumors [32]. Examination of autophagy-correlated proteins was performed, which manifested that SHK distinctly suppressed LC3II/I, but accelerated p62, elucidating that SHK boosted autophagy, as presented in figure 2f. In short, SHK repressed the SW480 cell proliferation.

### 3.3 MiR-545-3p inhibitor turns around the function of SHK on CRC cells

**Figure 3.**
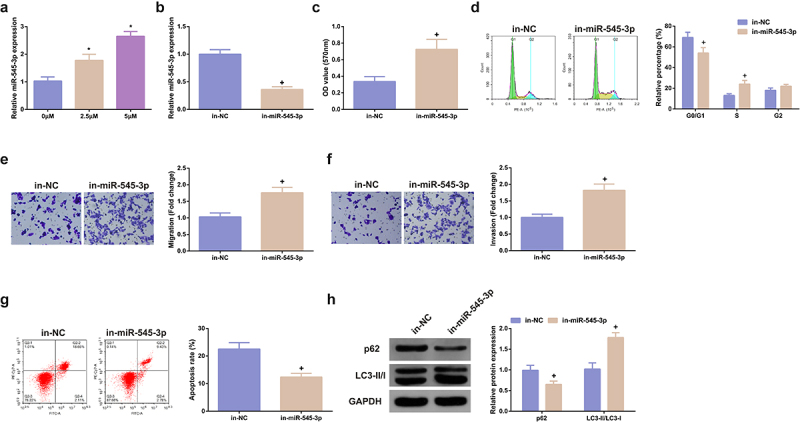
MiR-545-3p inhibitor turns around the action of SHK on CRC cells. (A) CRC cells were introduced with different concentrations of SHK medium to detect miR-545-3p in the cells. (B) Detecting miR-545-3p transfection efficiency via qPCR. (C) Determination of the impact of repressing miR-545-3p on cell viability was via MTT method. (D) Examination of the cell cycle variation after suppression of miR-545-3p was via Flow cytometry. (E, F) Test of cell migration and invasion after repressed miR-545-3p was via Transwell. (G) Examination of cell apoptosis after repression of miR-545-3p via Flow cytometry. (H) Analysis of autophagy-correlated protein was via Western blot, GAPDH presented as load control. N = 3; * *P* < 0.05 versus the 0 mg/ml concentration of SHK; + *P*< 0.05 versus the in-NC. The data in the figures were all measurement data in the form of mean ± SD.

MiR-545-3p’s impact on SHK-stimulated SW480 cells was figured out. Test of miR-545-3p in cells treating with different concentrations of SHK was conducted, clarifying that miR-545-3p was dramatically elevated dose dependently (Figure 3a). While the concentration was 5 μM, SHK distinctly facilitated miR-545-3p. Consequently, adoption of the concentration was as subsequent experiments. Subsequently, transfection of miR-545-3p inhibitor was into SHK-stimulated CRC, and miR-545-3p was successfully declined in SW480 cells (Figure 3b). MiR-545-3p inhibitor critically strengthened the cell viability (Figure 2c), the G0/G1 phase of cells was distinctively declined (Figure 2d), and the cell advancement was strengthened (Figure 2e-g). Additionally, the influence of miR-545-3p dysregulation on autophagy was explored. Repression of miR-545-3p distinctively elevated LC3II/I, but p62 was declined, elucidating that silence of miR-545-3p restrained autophagy, as proved in Figure 2h. In brief, repression of miR-545-3p effectively counteracted the influence of SHK on SW480 cells.

### 3.4 MiR-545-3p targets GNB1 in CRC cells

**Figure 4.**
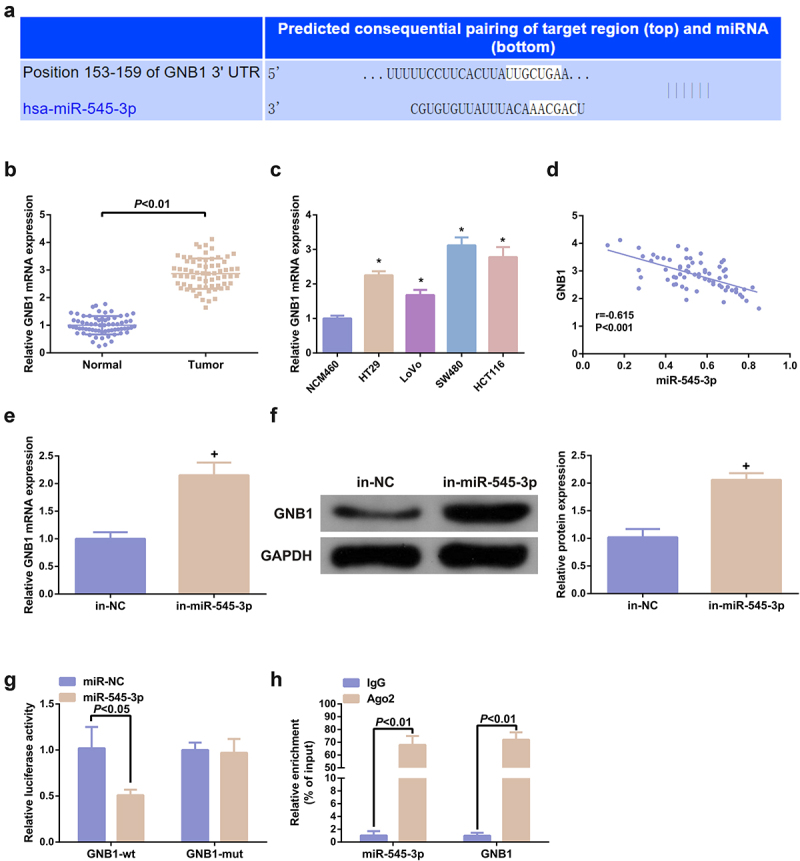
MiR-545-3p targets GNB1. (A) Sequence alignment was of miR-545-3p with GNB1 3ʹUTR-WT and GNB1 3ʹUTR-MUT. (BC) GNB1 mRNA in CRC and cell lines. (D) Pearson correlation analysis elucidated reverse association of miR-545-3p with GNB1 mRNA in the collected tumor tissue samples. (EF) GNB1 mRNA and protein in cells was elevated behind transfection of miR-545-3p inhibitor. (G) Elevated miR-545-3p in cells repressed the relative activity of GNB1 3ʹUTR-WT luciferase. (H) Test of CRC cells was via RNA immunoprecipitation with AGO2 antibody. The results of reverse transcription quantitative PCR clarified that AGO2 simultaneously enriched GNB1 mRNA and miR-545-3p. N = 3; * *P* < 0.05 versus the NCM460 cells; + *P*< 0.05 versus the in-NC. The data in the figures were all measurement data in the form of mean ± SD.

Prediction of miR-545-3p’s potential target genes was adopting TargetScan software, which elucidated that a putative binding site of miR-545-3p in the 3ʹUTR of GNB1 mRNA (Figure 4a). GNB1 was elevated in colorectal tumors and cell lines (Figure 4b, c). Additionally, miR-545-3p was reversely linked with GNB1 in the collected tissue samples (r = −0.615) (Figure 4d). Subsequently, silence of miR-545-3p was able to augment GNB1 mRNA and protein (Figure 4e, f). Elevated miR-545-3p declined the relative luciferase activity of GNB1 3ʹUTR-wt, but the relative luciferase activity of GNB1 3ʹUTR-mut was not impacted (Figure 4g). Furthermore, miR-545-3p directly interacted with GNB1 3ʹURT in SW480 cells. RIP test was also performed to verify this direct interaction. The results clarified that AGO2 simultaneously enriched GNB1 mRNA and miR-545-3p in SW480 cells (Figure 4h). In general, miR-545-3p targeted GNB1 in CRC cells.

### 3.5 Elevation of GNB1 turns around the anti-cancer action of SHK on CRC cells

**Figure 5.**
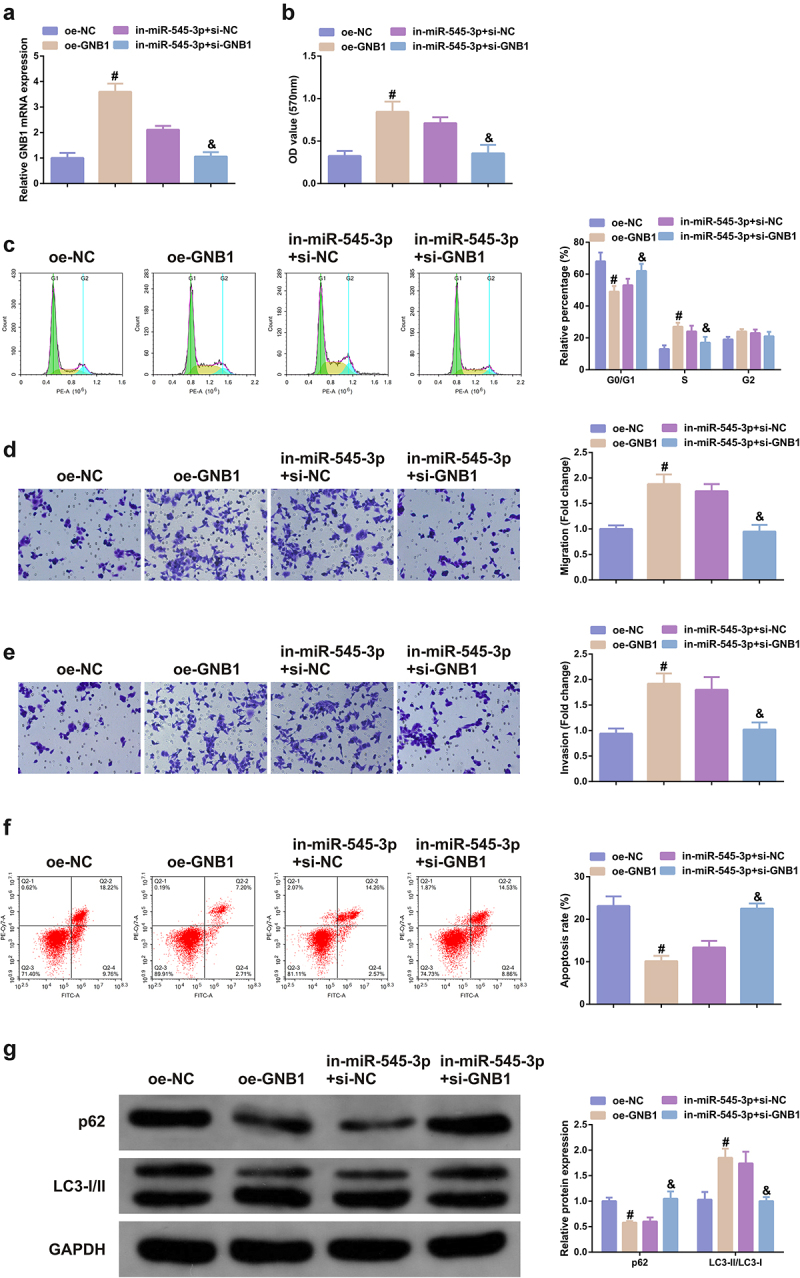
Elevation of GNB1 turns around the tumor suppressor effect of SHK on CRC cells. (A) Examination of the transfection efficiency of elevating GNB1 was via qPCR. (B) Determination of the impact of elevation of GNB1 on cell viability was via MTT method. (C) Detection of cell cycle variation after augmentation of GNB1 was via Flow cytometry. (D, E) Detection of the migration and invasion of cells after elevation of GNB1 was via Transwell. (F) Test of cell apoptosis after elevation of GNB1 was via Flow cytometry. (G) Analysis of autophagy-related protein was via Western blot, GAPDH presented as load control. N = 3; # *P*< 0.05 versus the oe-NC; & *P* < 0.05 versus the in-miR-545-3p + si-NC. The data in the figures were all measurement data in the form of mean ± SD.

The function of GNB1 on CRC cells was further analyzed. GNB1 was augmented in SHK-stimulated SW480 cells, and test of the transfection efficiency was performed (Figure 5a); Elevation of GNB1 augmented the advancement of SW480 cells with declined the apoptosis, autophagy and the cell quantities in G0/G1 phase (Figure 5b-f). Additionally, GNB1 was knocked down in cells with declined miR-545-3p, which manifested that GNB1 turned around repression of the miR-545-3p’s action on CRC cells (Figure 5a-f), elucidating that GNB1 mediated the impact of miR-545-3p on SW480 cells.

### 3.6 SHK represses the tumor progression

**Figure 6.**
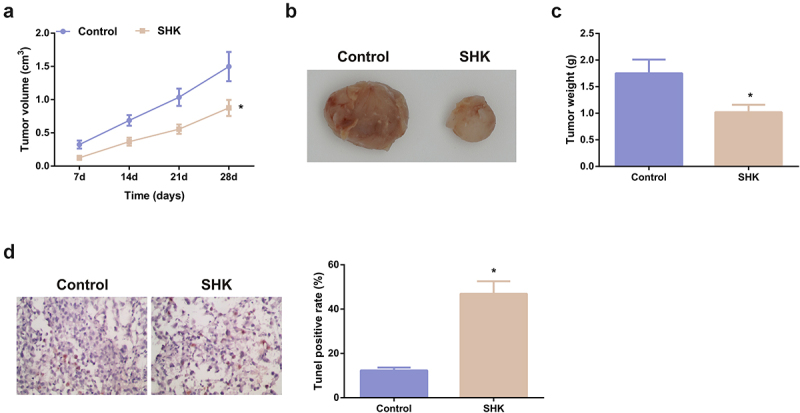
SHK represses tumor advancement. (A, B) The volume and tumor map of xenogeneic tumors. (C) The weight of the xenogeneic tumor. (D) Examination of tumor tissue apoptosis was with TUNEL staining. N = 6; *P < 0.05 versus the control. The data in the figures were all measurement data in the form of mean ± SD.

The modulation of SHK was explored. The volume and weight of the SHK were dramatically declined versus the control, as proved in Figure 6a -C. Besides, the apoptosis rate was distinctively elevated (Figure 6d). Consequently, SHK was available to repress the tumor progression in part via modulating the miR-545-3p/GNB1 axis.

## 4 Discussion

As is known to all, CRC is the most prevalent cancers in the world with the climbing morbidity year by year. The extremely poor prognosis, high mortality rate and high recurrence rate multiply elevated the difficulty of treatment [33]. natural plant ingredients with the merits of declined toxicity and declined cost have gradually become candidates for the treatment of CRC in the past few years [34]. In this study the role and mechanism of SHK in CRC were investigated. Based on the current results, it was found that SHK clearly repressed the proliferation, migration and invasion, but induced apoptosis and autophagy of CRC cells in a concentration-dependent manner. Meanwhile, it was also discovered that the restraining of SHK on cancer cells was achieved through miR-545-3p/GNB1 signaling axis, and *in vivo* experimental results further demonstrated SHK had clearly repressive effect on tumor formation.

SHK, the naphthoquinone compounds extracting from the roots of SHK, has been broadly explored for its anti-tumor and anti-inflammatory activities [35]. For instance, SHK represses cancer cell glycolysis via targeting tumor pyruvate kinase-M2 [36]. SHK ameliorates liver inflammation in diabetic Db/Db mice via Rho-kinase pathway [37]. Additionally, SHK has also exerted anti-cancer activity in CRC, that is, SHK stimulates apoptosis and autophagy in CRC via targeting galectin-1/JNK signaling axis [38]. CRC cells were treated with different concentrations of SHK, and functional experiments manifested, with the elevation of SHK concentration, the viability, migration and invasion abilities of the cells were apparently repressed, and the cells were organized in the G0/G1 phase, and the apoptosis level of the cells was also memorably elevated in a dose-dependent manner. Autophagy has been considered as a highly conservative metabolic process, which sustains homeostasis via recycling and degrading intracellular components to cope with the lack of nutrients or growth factors [39]. Multiple evidences clarifies that autophagy dysregulation is in constant inflammation and tumor cell proliferation, repressing or facilitating cancer progression [40]. Consequently, autophagy modulation is considered as a potential strategy for cancer treatment. In this study, referring to [41], it was learned that with the transition from LC3-I to LC3-II, the reduced expression of p62 could be regarded as a marker of autophagy. Through Western blot detection of LC3-II /LC3-I ratio and changes of p62, was discovered that SHK promoted the protein expression of p62 and the transformation of LC3-I to LC3-II, indicating that it induced autophagy of cells. In summary, the data manifested SHK repressed the proliferation, migration and invasion, but induced autophagy and apoptosis of CRC cells, thus destroying the process of CRC cells.

The latent mechanism of SHK’s repression on CRC cells was further explored. The anticancer effects of traditional Chinese medicines acting by targeting miRNAs have also been widely reported, for example, matrine-induced apoptosis of thyroid papillary carcinoma cells *in vitro* and restrains tumor growth *in vivo* by down-regulating miR-182-5p [42]. MiRNA is a vital factor in the occurrence of CRC and is able to be adopted as a brand-new potential biomarker for CRC diagnosis and cure [43]. For instance, miR-18a-5p is elevated in the blood of CRC patients as a potential biomarker for CRC diagnosis [44]. MiR-875-3p mitigates the CRC progression via negatively modulating polo-like kinase 1 (PLK1) [45]. In this study, firstly, the expression of miR-545-3p in clinical samples was detected, and it was found that miR-545-3p was clearly down-regulated in CRC patients, and the overall survival rate of CRC patients with reduced expression of miR-545-3p was poor, which was clearly associated with lymph node metastasis, distant metastasis and TNM stage. Then it was found that, SHK accelerated miR-545-3p dose-dependently. MiR-545-3p was repressed in SHK-treated cells, finding that repressed miR-545-3p turned around the suppression of SHK on the CRC progression, elucidating that miR-545-3p was supposed to be a downstream modulator of SHK impacting the CRC progress. The target of miR-545-3p was further figured out.

GNB1 is frequently associated with human neurological diseases. For instance, the novel GNB1 mutation destroyed the assembly and function of the G protein heterotrimers and led to overall human developmental delay [46]. GNB1 also exerts a crucial part in human cancer in the past few years. Furthermore, circular RNA_POLA2 accelerates the dryness of lung cancer cells via modulating the miR-326/GNB1 axis [47]. GNB1 is augmented in breast cancer (BC) and is positively associated with mammalian target of rapamycin. Furthermore, GNB1 is provided with a potential targeting in human breast cancer (HBC) therapy [48]. Within this research, GNB1 in CRC was augmented and was reversely linked with miR-545-3p;The elevation of GNB1 turned around the tumor suppressor effect of SHK on CRC cells, augmented the CRC cell advancement with declined G0/G1 phase cells, apoptosis and autophagy. Therefore, it was hypothesized that GNB1 might be the target of miR-545-3p. This conjecture was confirmed by dual-luciferase reporter gene and RIP assays. In brief, SHK mediated CRC progression via targeting miR-545-3p/GNB1 signaling axis.

Last but not least, miR-545-3p /GNB1 has been testified to reverse the therapeutic effects of SHK. However, it is not clear whether the mechanism has the same impacts *in vivo* experiments. Therefore, it is urgent to further study the molecular mechanism of SHK through miR-545-3p/GNB1 axis *in vivo*, which will be carried out in future experiments.

## 5 Conclusion

In conclusion, it is discovered that SHK restrains CRC cell viability, migration, and invasion in a dose-dependent manner. The study reveals for the first time the molecular mechanism in which SHK mediates CRC progression by targeting miR-545-3p/GNB1 signaling axis. This provides a theoretical basis for the future use of SHK as a novel therapeutic drug for CRC. It also provides a new perspective for the treatment of CRC.
